# Supplementary material for: Long-term maxillary anteroposterior changes following maxillary protraction with or without expansion: A meta-analysis and meta-regression
Source: PLoS One. 2021 Feb 22;16(2):e0247027. doi: 10.1371/journal.pone.0247027 (PMC7899359; doi:10.1371/journal.pone.0247027)
Supplement: S2 Table — (DOCX) [file pone.0247027.s002.docx]

| **S2 Table**  **List of included and excluded studies, with the corresponding reasons** | |
| --- | --- |
| Paper | Decision |
| Aloufi F, Preston CB, Zawawi KH (2012) Changes in the upper and lower pharyngeal airway spaces associated with rapid maxillary expansion. ISRN Dent 2012: 290964. | Excluded by title |
| Baratieri Cda L, Alves M, Jr., Mattos CT, Lau GW, Nojima LI, de Souza MM (2014) Transverse effects on the nasomaxillary complex one year after rapid maxillary expansion as the only intervention: a controlled study. Dental Press J Orthod 19: 79-87. | Excluded by title |
| Chang Y, Koenig LJ, Pruszynski JE, Bradley TG, Bosio JA, Liu D (2013) Dimensional changes of upper airway after rapid maxillary expansion: a prospective cone-beam computed tomography study. Am J Orthod Dentofacial Orthop 143: 462-70. | Excluded by title |
| Chen X, Liu D, Liu J, et al. (2015) Three-Dimensional Evaluation of the Upper Airway Morphological Changes in Growing Patients with Skeletal Class III Malocclusion Treated by Protraction Headgear and Rapid Palatal Expansion: A Comparative Research. PLoS One 10: e0135273. | Excluded by title |
| Hiyama S, Suda N, Ishii-Suzuki M, et al. (2002) Effects of maxillary protraction on craniofacial structures and upper-airway dimension. Angle Orthod 72: 43-7. | Excluded by title |
| Isik F, Sayinsu K, Nalbantgil D, Arun T (2005) A comparative study of dental arch widths: extraction and non-extraction treatment. Eur J Orthod 27: 585-9. | Excluded by title |
| El H, Palomo JM (2014) Three-dimensional evaluation of upper airway following rapid maxillary expansion: a CBCT study. Angle Orthod 84: 265-73. | Excluded by title |
| Guest SS, McNamara JA, Jr., Baccetti T, Franchi L (2010) Improving Class II malocclusion as a side-effect of rapid maxillary expansion: a prospective clinical study. Am J Orthod Dentofacial Orthop 138: 582-91. | Excluded by title |
| Hiyama S, Suda N, Ishii-Suzuki M, et al. (2002) Effects of maxillary protraction on craniofacial structures and upper-airway dimension. Angle Orthod 72: 43-7. | Excluded by title |
| Kaygisiz E, Tuncer BB, Yuksel S, Tuncer C, Yildiz C (2009) Effects of maxillary protraction and fixed appliance therapy on the pharyngeal airway. Angle Orthod 79: 660-7. | Excluded by title |
| Kilinc AS, Arslan SG, Kama JD, Ozer T, Dari O (2008) Effects on the sagittal pharyngeal dimensions of protraction and rapid palatal expansion in Class III malocclusion subjects. Eur J Orthod 30: 61-6. | Excluded by title |
| Kurt G, Altug-Atac AT, Atac MS, Karasu HA (2010) Changes in nasopharyngeal airway following orthopedic and surgically assisted rapid maxillary expansion. J Craniofac Surg 21: 312-7. | Excluded by title |
| Langer MR, Itikawa CE, Valera FC, Matsumoto MA, Anselmo-Lima WT (2011) Does rapid maxillary expansion increase nasopharyngeal space and improve nasal airway resistance? Int J Pediatr Otorhinolaryngol 75: 122-5. | Excluded by title |
| Li L, Qi S, Wang H, Ren S, Ban J (2015) [Cone-beam CT evaluation of nasomaxillary complex and upper airway following rapid maxillary expansion]. Zhonghua Kou Qiang Yi Xue Za Zhi 50: 403-7. | Excluded by title |
| Litton SF, Ackermann LV, Isaacson RJ, Shapiro BL (1970) A genetic study of Class 3 malocclusion. Am J Orthod 58: 565-77. | Excluded by title |
| Liu Z, McGrath C, H?gg U (2009) The impact of malocclusion/orthodontic treatment need on the quality of life. A systematic review. Angle Orthod 79: 585-91. | Excluded by title |
| Melsen B, Melsen F (1982) The postnatal development of the palatomaxillary region studied on human autopsy material. Am J Orthod 82: 329-42. | Excluded by title |
| Mucedero M, Baccetti T, Franchi L, Cozza P (2009) Effects of maxillary protraction with or without expansion on the sagittal pharyngeal dimensions in Class III subjects. Am J Orthod Dentofacial Orthop 135: 777-81. | Excluded by title |
| Phatouros A, Goonewardene MS (2008) Morphologic changes of the palate after rapid maxillary expansion: a 3-dimensional computed tomography evaluation. Am J Orthod Dentofacial Orthop 134: 117-24. | Excluded by title |
| Ribeiro AN, de Paiva JB, Rino-Neto J, Illipronti-Filho E, Trivino T, Fantini SM (2012) Upper airway expansion after rapid maxillary expansion evaluated with cone beam computed tomography. Angle Orthod 82: 458-63. | Excluded by title |
| Silverman SL (2009) From randomized controlled trials to observational studies. Am J Med 122: 114-20. | Excluded by title |
| Smith T, Ghoneima A, Stewart K, et al. (2012) Three-dimensional computed tomography analysis of airway volume changes after rapid maxillary expansion. Am J Orthod Dentofacial Orthop 141: 618-26. | Excluded by title |
| Zeng J, Gao X (2013) A prospective CBCT study of upper airway changes after rapid maxillary expansion. Int J Pediatr Otorhinolaryngol 77: 1805-10. | Excluded by title |
| Zhao Y, Nguyen M, Gohl E, Mah JK, Sameshima G, Enciso R (2010) Oropharyngeal airway changes after rapid palatal expansion evaluated with cone-beam computed tomography. Am J Orthod Dentofacial Orthop 137: S71-8. | Excluded by title |
| Almuzian M, Almukhtar A, Ulhaq A, Alharbi F (2019) 3D effects of a bone-anchored intra-oral protraction in treating class III growing patient: a pilot study. 20: 37. | Excluded by title |
| Angelieri F, Ruellas AC, Yatabe MS, et al. (2017) Zygomaticomaxillary suture maturation: Part II-The influence of sutural maturation on the response to maxillary protraction. Orthod Craniofac Res 20: 152-63. | Excluded by title |
| Iwasaki T, Saitoh I, Takemoto Y, et al. (2013) Tongue posture improvement and pharyngeal airway enlargement as secondary effects of rapid maxillary expansion: a cone-beam computed tomography study. Am J Orthod Dentofacial Orthop 143: 235-45. | Excluded by abstract |
| Elnagar MH, Elshourbagy E, Ghobashy S, Khedr M, Evans CA (2016) Comparative evaluation of 2 skeletally anchored maxillary protraction protocols. Am J Orthod Dentofacial Orthop 150: 751-62. | Excluded by abstract |
| Almuzian M, McConnell E, Darendeliler MA, Alharbi F, Mohammed H (2018) The effectiveness of alternating rapid maxillary expansion and constriction combined with maxillary protraction in the treatment of patients with a class III malocclusion: a systematic review and meta-analysis. J Orthod 45: 250-59. | Excluded by full-text  - systematic review |
| Bucci R, D'Anto V, Rongo R, Valletta R, Martina R, Michelotti A (2016) Dental and skeletal effects of palatal expansion techniques: a systematic review of the current evidence from systematic reviews and meta-analyses. J Oral Rehabil 43: 543-64. | Excluded by full-text  - systematic review |
| Cordasco G, Matarese G, Rustico L, et al. (2014) Efficacy of orthopedic treatment with protraction facemask on skeletal Class III malocclusion: a systematic review and meta-analysis. Orthod Craniofac Res 17: 133-43. | Excluded by full-text  - systematic review |
| Foersch M, Jacobs C, Wriedt S, Hechtner M, Wehrbein H (2015) Effectiveness of maxillary protraction using facemask with or without maxillary expansion: a systematic review and meta-analysis. Clin Oral Investig 19: 1181-92. | Excluded by full-text  - systematic review |
| Kim JH, Viana MA, Graber TM, Omerza FF, BeGole EA (1999) The effectiveness of protraction face mask therapy: a meta-analysis. Am J Orthod Dentofacial Orthop 115: 675-85. | Excluded by full-text  - systematic review |
| Lin Y, Guo R, Hou L, Fu Z, Li W (2018) Stability of maxillary protraction therapy in children with Class III malocclusion: a systematic review and meta-analysis. Clin Oral Investig 22: 2639-52. | Excluded by full-text  - systematic review |
| Pithon MM, Santos NL, Santos CR, et al. (2016) Is alternate rapid maxillary expansion and constriction an effective protocol in the treatment of Class III malocclusion? A systematic review. Dental Press J Orthod 21: 34-42. | Excluded by full-text  - systematic review |
| Polito I, Martina R, Michelotti A, Woon SC, Thiruvenkatachari B (2017) Early orthodontic treatment for Class III malocclusion: A systematic review and meta-analysis. J Oral Rehabil 151: 28-52. | Excluded by full-text  - systematic review |
| Rodriguez de Guzman-Barrera J, Saez Martinez C, Boronat-Catala M, et al. (2017) Effectiveness of interceptive treatment of class III malocclusions with skeletal anchorage: A systematic review and meta-analysis. PLoS One 12: e0173875. | Excluded by full-text  - systematic review |
| Rongo R, D'Anto V, Bucci R (2017) Skeletal and dental effects of Class III orthopaedic treatment: a systematic review and meta-analysis. 44: 545-62. | Excluded by full-text  - systematic review |
| Woon SC, Thiruvenkatachari B (2017) Early orthodontic treatment for Class III malocclusion: A systematic review and meta-analysis. Am J Orthod Dentofacial Orthop 151: 28-52. | Excluded by full-text  - systematic review |
| Zhang W, Qu HC, Yu M, Zhang Y (2015) The Effects of Maxillary Protraction with or without Rapid Maxillary Expansion and Age Factors in Treating Class III Malocclusion: A Meta-Analysis. PLoS One 10: e0130096. | Excluded by full-text  - systematic review |
| Isci D, Turk T, Elekdag-Turk S (2010) Activation-deactivation rapid palatal expansion and reverse headgear in Class III cases. Eur J Orthod 32: 706-15. | Excluded by full-text  - Full texts were not eligible |
| De Clerck H, Cevidanes L, Baccetti T (2010) Dentofacial effects of bone-anchored maxillary protraction: a controlled study of consecutively treated Class III patients. Am J Orthod Dentofacial Orthop 138: 577-81. | Excluded by full-text  - Full texts were not eligible |
| Auconi P, Scazzocchio M, Cozza P, McNamara JA, Jr., Franchi L (2015) Prediction of Class III treatment outcomes through orthodontic data mining. Eur J Orthod 37: 257-67. | Excluded by full-text  - Full texts were not eligible |
| Sar C, Sahinoglu Z, Ozcirpici AA, Uckan S (2014) Dentofacial effects of skeletal anchored treatment modalities for the correction of maxillary retrognathia. Am J Orthod Dentofacial Orthop 145: 41-54. | Excluded by full-text  - Full texts were not eligible |
| Basciftci FA, Karaman AI (2002) Effects of a modified acrylic bonded rapid maxillary expansion appliance and vertical chin cap on dentofacial structures. Angle Orthod 72: 61-71. | Excluded by full-text  - Full texts were not eligible |
| Cameron CG, Franchi L, Baccetti T, McNamara JA, Jr. (2002) Long-term effects of rapid maxillary expansion: a posteroanterior cephalometric evaluation. Am J Orthod Dentofacial Orthop 121: 129-35; quiz 93. | Excluded by full-text  - Full texts were not eligible |
|  |  |
| Ngan P, Wilmes B, Drescher D, Martin C, Weaver B, Gunel E (2015) Comparison of two maxillary protraction protocols: tooth-borne versus bone-anchored protraction facemask treatment. Prog Orthod 16: 26. | Excluded by full-text  - no untreated CIII control |
| Onem Ozbilen E, Yilmaz HN, Kucukkeles N (2019) Comparison of the effects of rapid maxillary expansion and alternate rapid maxillary expansion and constriction protocols followed by facemask therapy. Korean J Orthod 49: 49-58. | Excluded by full-text  - no untreated CIII control |
| Chang JY, McNamara JA, Jr., Herberger TA (1997) A longitudinal study of skeletal side effects induced by rapid maxillary expansion. Am J Orthod Dentofacial Orthop 112: 330-7. | Excluded by full-text  - no untreated CIII control |
| Seiryu M, Ida H, Mayama A, et al. (2019) A comparative assessment of orthodontic treatment outcomes of mild skeletal Class III malocclusion between facemask and facemask in combination with a miniscrew for anchorage in growing patients: A single-center, prospective randomized controlled trial. Angle Orthod. | Excluded by full-text  - no untreated CIII control |
| Wilmes B, Ngan P, Liou EJ, Franchi L, Drescher D (2014) Early class III facemask treatment with the hybrid hyrax and Alt-RAMEC protocol. J Clin Orthod 48: 84-93. | Excluded by full-text  - no untreated CIII control |
| Geran RG, McNamara JA, Jr., Baccetti T, Franchi L, Shapiro LM (2006) A prospective long-term study on the effects of rapid maxillary expansion in the early mixed dentition. Am J Orthod Dentofacial Orthop 129: 631-40. | Excluded by full-text  - no untreated CIII control |
| Ge YS, Liu J, Chen L, Han JL, Guo X (2012) Dentofacial effects of two facemask therapies for maxillary protraction. Angle Orthod 82: 1083-91. | Excluded by full-text  - no untreated CIII control |
| Pinheiro FH, Garib DG, Janson G, Bombonatti R, de Freitas MR (2014) Longitudinal stability of rapid and slow maxillary expansion. Dental Press J Orthod 19: 70-7. | Excluded by full-text  - no untreated CIII control |
| Kurt G, Uysal T, Yagci A (2010) Soft and hard tissue profile changes after rapid maxillary expansion and face mask therapy. World J Orthod 11: e10-8. | Excluded by full-text  - no untreated CIII control |
| Williams MD, Sarver DM, Sadowsky PL, Bradley E (1997) Combined rapid maxillary expansion and protraction facemask in the treatment of Class III malocclusions in growing children: a prospective long-term study. Semin Orthod 3: 265-74. | Excluded by full-text  - no untreated CIII control |
| Shanker S, Ngan P, Wade D, et al. (1996) Cephalometric A point changes during and after maxillary protraction and expansion. Am J Orthod Dentofacial Orthop 110: 423-30. | Excluded by full-text  - no untreated CIII control |
| Wolfe SM, Araujo E, Behrents RG, Buschang PH (2011) Craniofacial growth of Class III subjects six to sixteen years of age. Angle Orthod 81: 211-6. | Excluded by full-text  - no untreated CIII control |
| Ngan P, Yiu C, Hu A, Hagg U, Wei SH, Gunel E (1998) Cephalometric and occlusal changes following maxillary expansion and protraction. Eur J Orthod 20: 237-54. | Excluded by full-text  - not clinical controlled trial |
| O'Grady PW, McNamara JA, Jr., Baccetti T, Franchi L (2006) A long-term evaluation of the mandibular Schwarz appliance and the acrylic splint expander in early mixed dentition patients. Am J Orthod Dentofacial Orthop 130: 202-13. | Excluded by full-text  - not clinical controlled trial |
| Ngan P, Hagg U, Yiu C, Merwin D, Wei SH (1996) Treatment response to maxillary expansion and protraction. Eur J Orthod 18: 151-68. | Excluded by full-text  - not clinical controlled trial |
| Kapust AJ, Sinclair PM, Turley PK (1998) Cephalometric effects of face mask/expansion therapy in Class III children: a comparison of three age groups. Am J Orthod Dentofacial Orthop 113: 204-12. | Excluded by full-text  - not clinical controlled trial |
| McNamara JA, Jr., Baccetti T, Franchi L, Herberger TA (2003) Rapid maxillary expansion followed by fixed appliances: a long-term evaluation of changes in arch dimensions. Angle Orthod 73: 344-53. | Excluded by full-text  - not clinical controlled trial |
| Franchi L, Baccetti T, McNamara JA (2004) Postpubertal assessment of treatment timing for maxillary expansion and protraction therapy followed by fixed appliances. Am J Orthod Dentofacial Orthop 126: 555-68. | Excluded by full-text  - not clinical controlled trial |
| Pinheiro FH, Garib DG, Janson G, Bombonatti R, de Freitas MR (2014) Longitudinal stability of rapid and slow maxillary expansion. Dental Press J Orthod 19: 70-7. | Excluded by full-text  -not CIII malocclusion |
| Liou EJ, Tsai WC (2005) A new protocol for maxillary protraction in cleft patients: repetitive weekly protocol of alternate rapid maxillary expansions and constrictions. Cleft Palate Craniofac J 42: 121-7. | Excluded by full-text  - Patients with craniofacial anomaly |
| Delaire J, Verdon P, Lumineau JP, Cherga-Negrea A, Talmant J, Boisson M (1972) [Some results of extra-oral tractions with front-chin rest in the orthodontic treatment of class 3 maxillomandibular malformations and of bony sequelae of cleft lip and palate]. Rev Stomatol Chir Maxillofac 73: 633-42. | Excluded by full-text  - Patients with craniofacial anomaly |
| Baccetti T, Franchi L, Mucedero M, Cozza P (2010) Treatment and post-treatment effects of facemask therapy on the sagittal pharyngeal dimensions in Class III subjects. Eur J Orthod 32: 346-50. | Excluded by full-text  - less than 6 months of follow-up. |
| Akkaya S, Lorenzon S, Ucem TT (1999) A comparison of sagittal and vertical effects between bonded rapid and slow maxillary expansion procedures. Eur J Orthod 21: 175-80. | Excluded by full-text  - less than 6 months of follow-up. |
| Akin M, Ucar FI, Chousein C, Sari Z (2015) Effects of chincup or facemask therapies on the orofacial airway and hyoid position in Class III subjects. J Orofac Orthop 76: 520-30. | Included |
| Balos Tuncer B, Ulusoy C, Tuncer C, Turkoz C, Kale Varlik S (2015) Effects of reverse headgear on pharyngeal airway in patients with different vertical craniofacial features. Braz Oral Res 29. | Included |
| Chen XH, Xie XQ (2012) [The effect of two different methods of rapid maxillary expansion on treatment results of skeletal Class III malocclusion patients with maxillary protraction in early permanent dentition]. Shanghai Kou Qiang Yi Xue 21: 580-3. | Included |
| Chong YH, Ive JC, Artun J (1996) Changes following the use of protraction headgear for early correction of Class III malocclusion. Angle Orthod 66: 351-62. | Included |
| Cozza P, Baccetti T, Mucedero M, Pavoni C, Franchi L (2010) Treatment and posttreatment effects of a facial mask combined with a bite-block appliance in Class III malocclusion. Am J Orthod Dentofacial Orthop 138: 300-10. | Included |
| Kilicoglu H, Kirlic Y (1998) Profile changes in patients with class III malocclusions after Delaire mask therapy. Am J Orthod Dentofacial Orthop 113: 453-62. | Included |
| Mandall N, DiBiase A, Littlewood S, Nute S, Stivaros N, et al. (2010) Is early Class III protraction facemask treatment effective? A multicentre, randomized, controlled trial: 15-month follow-up. J Orthod 37: 149-161. | Included |
| Mandall N, Cousley R, DiBiase A, et al. (2016) Early class III protraction facemask treatment reduces the need for orthognathic surgery: a multi-centre, two-arm parallel randomized, controlled trial. J Orthod 43: 164-75. | Included |
| Ucem TT, Ucuncu N, Yuksel S (2004) Comparison of double-plate appliance and facemask therapy in treating Class III malocclusions. Am J Orthod Dentofacial Orthop 126: 672-9. | Included |
| Vaughn GA, Mason B, Moon HB, Turley PK (2005) The effects of maxillary protraction therapy with or without rapid palatal expansion: a prospective, randomized clinical trial. Am J Orthod Dentofacial Orthop 128: 299-309. | Included |
| Kajiyama K, Murakami T, Suzuki A (2004) Comparison of orthodontic and orthopedic effects of a modified maxillary protractor between deciduous and early mixed dentitions. Am J Orthod Dentofacial Orthop 126: 23-32. | Included |
| Masucci C, Franchi L, Defraia E, Mucedero M, Cozza P, Baccetti T (2011) Stability of rapid maxillary expansion and facemask therapy: a long-term controlled study. Am J Orthod Dentofacial Orthop 140: 493-500. | Included |
| Masucci C, Franchi L, Giuntini V, Defraia E (2014) Short-term effects of a modified Alt-RAMEC protocol for early treatment of Class III malocclusion: a controlled study. Orthod Craniofac Res 17: 259-69. | Included |
| Sar C, Arman-Ozcirpici A, Uckan S, Yazici AC (2011) Comparative evaluation of maxillary protraction with or  without skeletal anchorage. Am J Orthod Dentofacial Orthop 139: 636-49. | Included |
| Westwood PV, McNamara JA, Jr., Baccetti T, Franchi L, Sarver DM (2003) Long-term effects of Class III treatment with rapid maxillary expansion and facemask therapy followed by fixed appliances. Am J Orthod Dentofacial Orthop 123: 306-20. | Included |
| Xu B, Lin J (2001) [The orthopedic treatment of skeletal class III malocclusion with maxillary protraction therapy]. Zhonghua Kou Qiang Yi Xue Za Zhi 36: 401-3. | Included |
| Yuksel S, Ucem TT, Keykubat A (2001) Early and late facemask therapy. Eur J Orthod 23: 559-68. | Included |
